# Streptococcal Bacteremia and Toxic Shock Syndrome: A Rare Etiology Requiring Prompt Diagnosis

**DOI:** 10.7759/cureus.43944

**Published:** 2023-08-22

**Authors:** Colleen Achong, Tutul Chowdhury, Fareeza Mustafa, David Smith, Harry Moussouris

**Affiliations:** 1 Internal Medicine, One Brooklyn Health, New York, USA; 2 Internal Medicine, Interfaith Medical Center, New York, USA; 3 Intensive Care Unit, One Brooklyn Health, New York, USA; 4 Pathology, One Brooklyn Health, New York, USA

**Keywords:** cytokine release, intravenous immunoglobulin, bullous impetigo, streptococcal bacteremia, toxic shock syndrome (tss)

## Abstract

Bullous impetigo leading to streptococcal toxic shock syndrome (STSS) immune activation and massive cytokine release is a rare condition. It has a significant mortality rate, which calls for quick diagnosis, early suspicion, and integrated treatment. Herein, we discuss the case of a 66-year-old man who underwent evaluation for leg swelling before quickly going into shock and experiencing respiratory failure, which necessitated invasive mechanical intubation. *Streptococcus pyogenes* was identified by blood culture, and STSS was identified. Recommended antibiotics, intravenous (IV) immunoglobulin, and fluids made up the treatment regimen. In this case, the streptococcal infection deteriorated very quickly, and there was a rare relationship with bullous impetigo, which led to shock and respiratory failure. This case sheds lights on the need of having an early suspicion of this syndrome when a diabetic patient develops a skin lesion. A prompt diagnosis is necessary.

## Introduction

*Streptococcus pyogenes* (also known as group A *Streptococcus *(GAS)) is a group of Gram-positive bacteria, known to cause a range of infections from benign tonsillitis, pharyngitis, and impetigo to post-acute infection sequelae, such as glomerulonephritis, and to severe invasive diseases, such as septicemia, necrotizing fasciitis, and streptococcal toxic shock syndrome (STSS). Repeated infections can trigger autoimmune response leading to rheumatic fever and rheumatic heart disease. Risk factors for invasive GAS infection include minor trauma, non-steroidal anti-inflammatory drug (NSAID) use, surgical procedures, gynecological procedures, HIV infection, intravenous (IV) drug use, viral infections like influenza, varicella, age (more than 65 years), homelessness, burns, obesity, peripheral vascular disease (PVD), diabetes mellitus (DM), cardiac disease, and steroid use [1,2,3]. Here, we present a rare case of bullous impetigo leading to TSS, caused by GAS.

## Case presentation

We have a 66-year-old male with a past medical history of chronic atrial fibrillation, cerebral vascular accident (CVA), DM, hyperlipidemia, and hypertension who was brought to the emergency department due to back pain. The patient also endorsed swelling to left leg, resulting from being hit by his child's scooter four days prior to admission.

On admission, the patient was awake, alert, and oriented to person and place but became progressively disoriented on the morning of the second day due to toxic shock and acute respiratory failure. The patient was placed on high flow. Then, after two hours, a central line was placed, and the patient was intubated due to worsening respiratory failure. On the third day of admission, the patient started having a few bullous lesions on the left leg that was already edematous; as the disease progressed, the bullous lesions expanded to 25% of the skin. This patient was treated for the toxic formation with broad-spectrum antibiotics, deescalating gradually. Due to the clinical presentation of STSS, the patient was managed with IV immunoglobulin. Vitals on presentation were as follows: blood pressure 122/78, pulse 91, temperature 36.5 °C (97.7 °F), respiratory rate 18, height 6' 3" (1.905 m), weight 325 lb (147.4 kg), and SpO_2_ 98%. Laboratory results on admission are illustrated in Table 1.

**Table 1:** Laboratory results on admission

Test	Values	Reference range and units
White blood cell (WBC)	13.5	4.5-11.0 10^3^/uL
Hemoglobin (Hb)	13.4	11.0-15.0 g/dL
Mean corpuscular volume (MCV)	87.3	80-100 fL
Platelets	196	130-400 10^3^/uL
Blood urea nitrogen (BUN)	38	7.0-18.7 mg/dL
Creatinine	2.1	0.57-1.11 mg/dL
Estimated glomerular filtration rate (eGFR)	34.1	≥90.0
Sodium (Na)	140	136-145 mmol/L
Potassium (K)	3.5	3.5-5.1 mmol/L
Carbon dioxide (CO_2_)	21	22-29 mmol/L
Anion gap	14	8-12
Total bilirubin	3.4	0.2-1.2 mg/dL
Alanine transaminase (ALT)	44	10-55 U/L
Aspartate aminotransferase (AST)	160	5-34 U/L
Alkaline phosphatase (ALP)	105	40-150 U/L
Albumin	3.1	3.5-5.2 g/dL
Phosphorous	2.6	2.3-4.7 mg/dl
Calcium (Ca)	9.0	8.4-10.2 mg/dL
Brain natriuretic peptide (BNP)	93	10-100 pg/ml
Lactate	4.1	0.50-1.90 mmol/L
High-sensitivity troponin	100	0-17 ng/ml
Prothrombin time (PT)	18	9.8-13.4 sec
International normalized ratio (INR)	1.5	0.85-1.15
Partial thromboplastin time (PTT)	32.5	24.9-35.9 sec
Creatine kinase total	3377	30.0-200.0 U/L
Hemoglobin A1c	9.3	4.8-5.6%
C-reactive protein (CRP)		0.5-1 mg/dl
Blood culture	*Streptococcus* species	Negative

**Figure 1:**
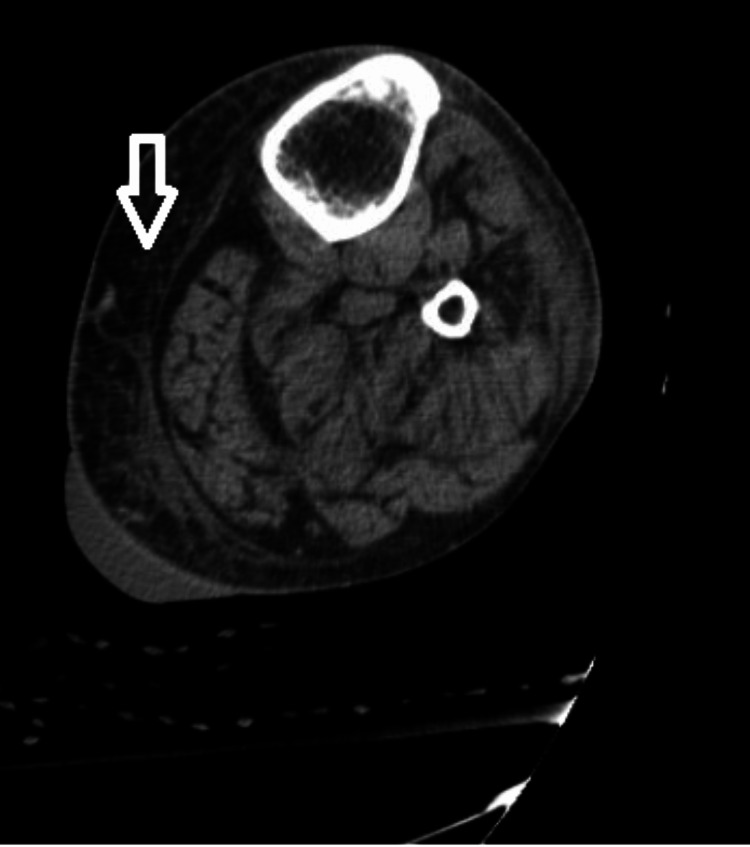
CT scan of the left leg illustrating subcutaneous edema of the lower leg (white arrow) CT: computed tomography

Doppler scan of the left leg was performed, which illustrated subcutaneous edema of the lower leg, consistent with cellulitis (Figure 1). A fluid collection within the skin at the calf region was also appreciated correlated to his blister. Arterial doppler done at the bedside showed dorsalis pedis and posterior tibial biphasic flow.

**Figure 2:**
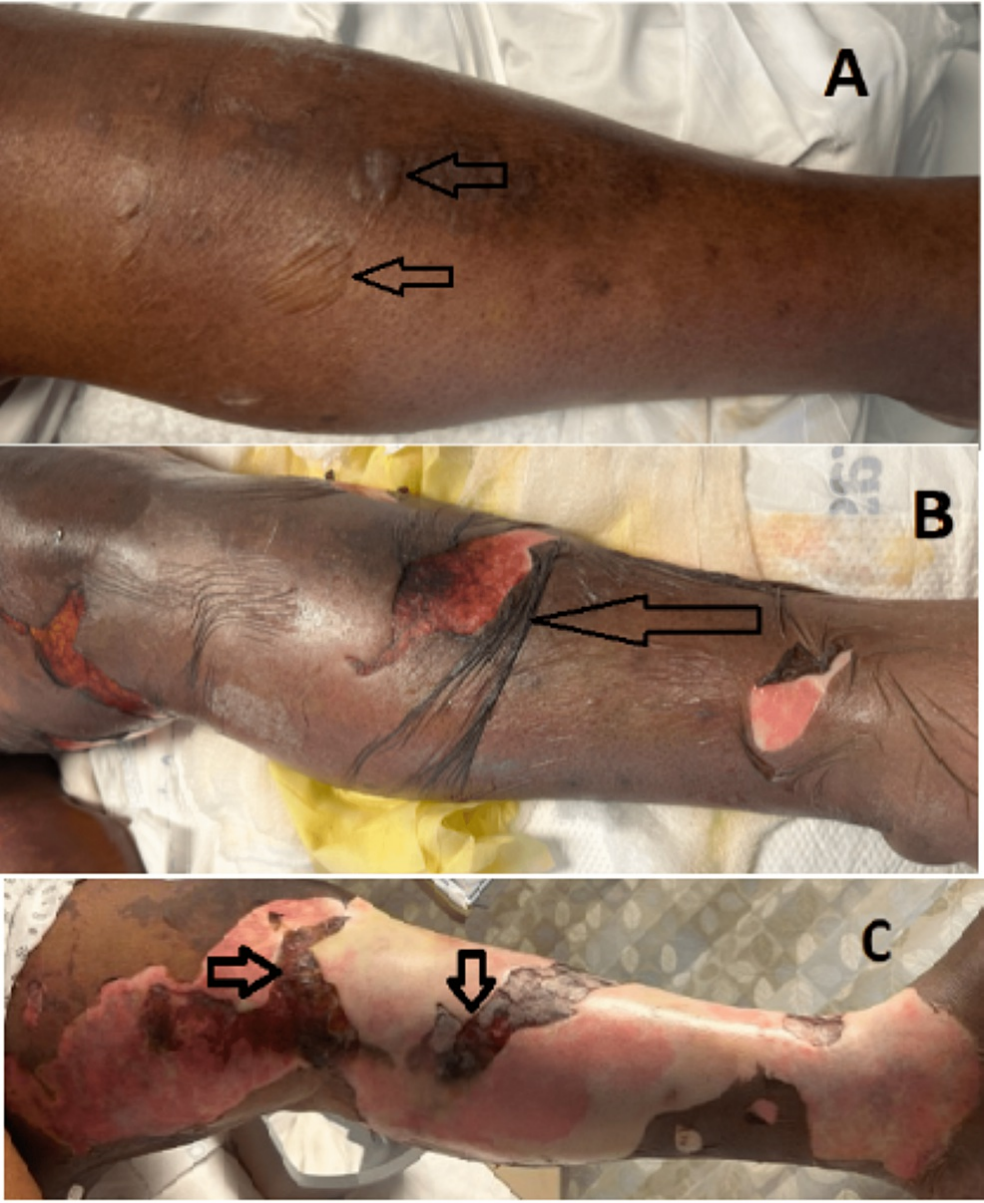
Left leg bullous formation progression to eschar. A, Day 1; B, Day 3; C, Day 5 This figure demonstrates left leg bullous formation progressing to skin sloughing and denuded skin with eschar.

**Figure 3:**
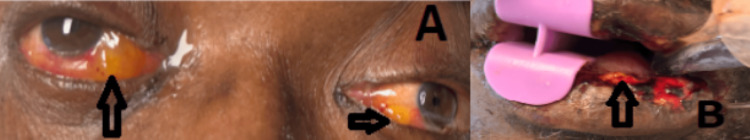
Mucous membrane involvement with the drainage of yellow thin fluid consistent with Streptococcus pyogenes discharge A: mucosal discharge with drainage of yellow fluid consistent with *Streptococcus pyogenes* in the ocular membrane. B: mucosal discharge from the upper and lower lip

Broad-spectrum antibiotics were instituted, de-escalating with sensitivity as indicated. On the third day, the patient started developing bullous lesions, progressing to about 28% of the lower extremities with the involvement of the bilateral upper extremities, abdomen, chest, lips, and gluteal folds (Figures 2, 3).

**Figure 4:**
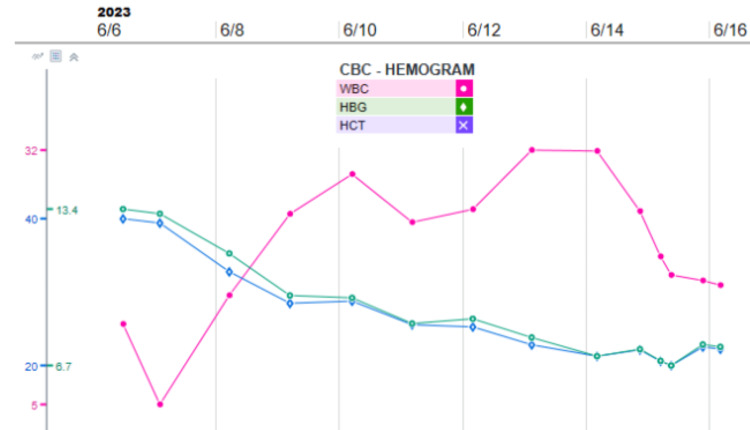
Hemoglobin (HGB), white blood cell (WBC), and hematocrit (HCT) trend during hospitalization

**Figure 5:**
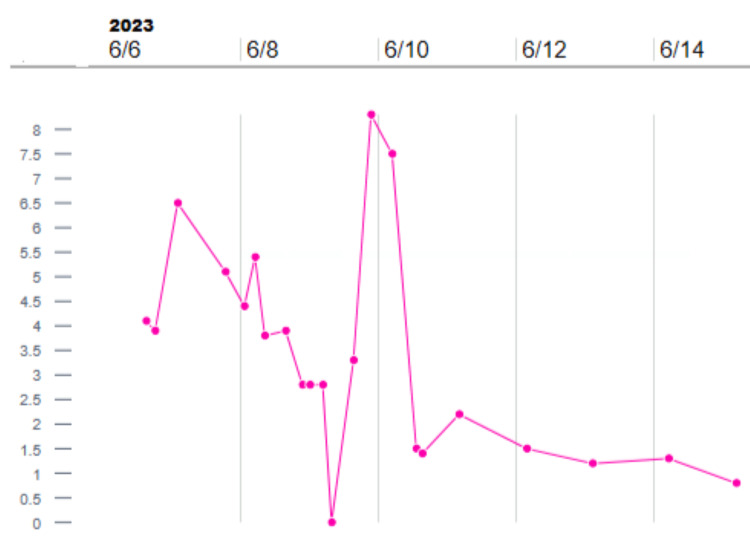
Lactate trend during hospitalization

**Figure 6:**
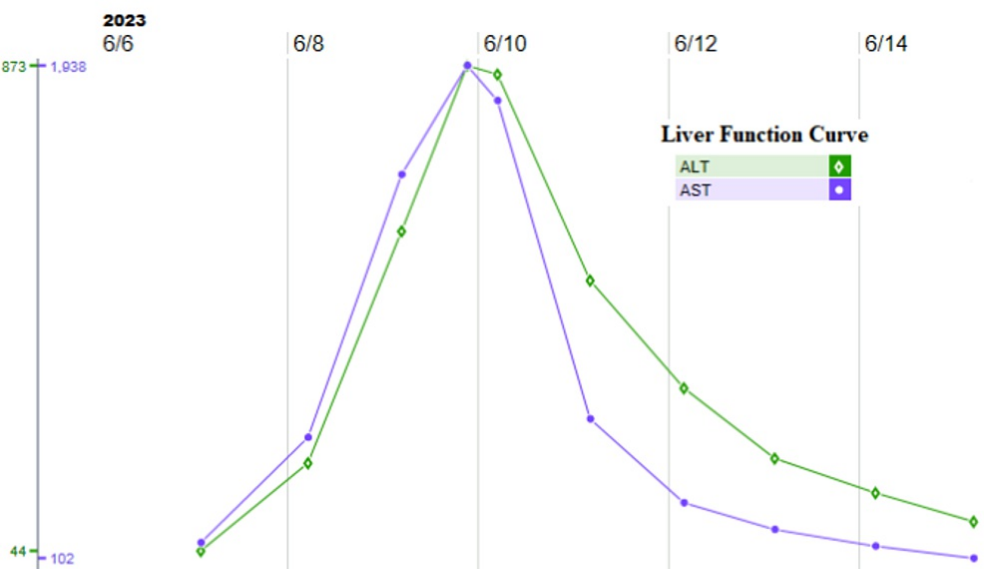
Liver transaminase trend during hospitalization ALT: alanine transaminase; AST: aspartate transaminase

Leukocytosis with lactic acidosis was evident in the lab work with deranged liver functions, while blood culture was growing *S. pyogenes *(Figure 4). Figure 5 and Figure 6 present the lactate trend and Liver transaminase trend during hospitalization, respectively.

**Figure 7:**
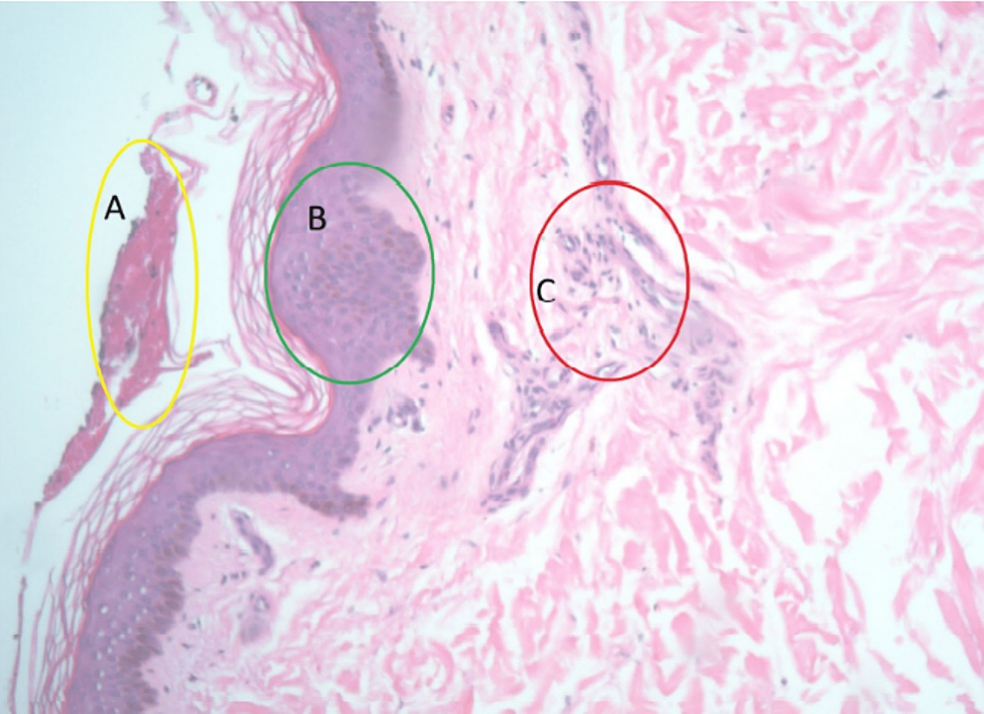
Biopsy image: A, some surface blood and inflammation; B, reactive surface squamous epithelium; C, focus of the dermal perivascular inflammation

**Figure 8:**
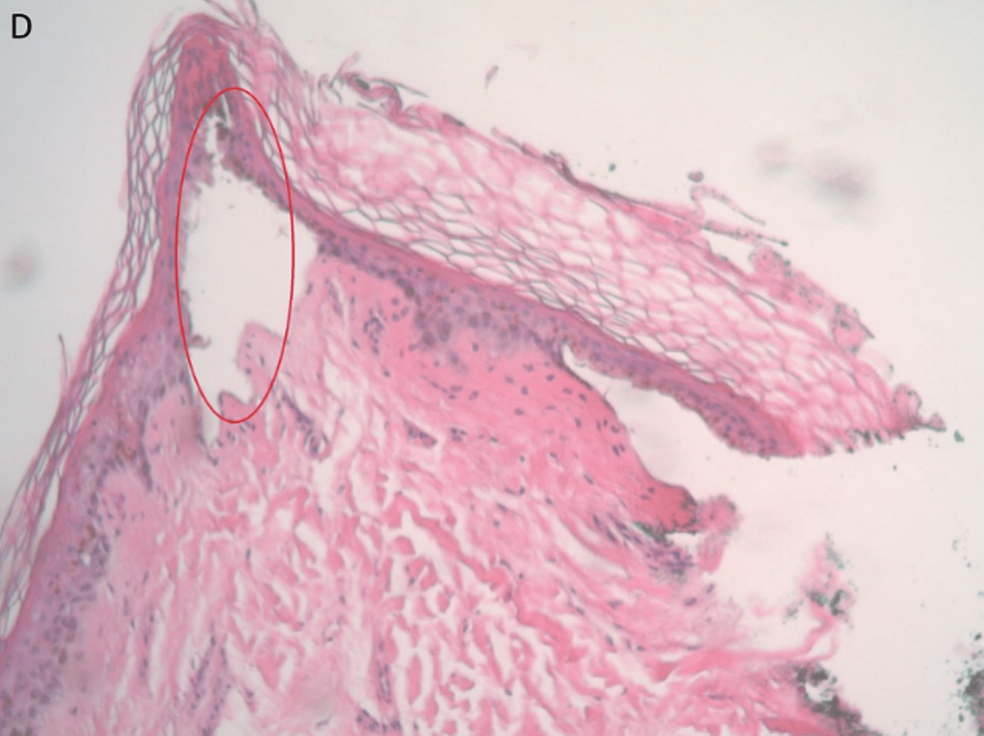
Biopsy image: D, bulla of the bullous impetigo histology

In the setting of septic shock, the patient was given hydrocortisone and fludrocortisone. An infectious disease specialist was consulted, and the antibiotic was changed to clindamycin and ceftriaxone. IV clindamycin was discontinued after the fifth day, and ceftriaxone was continued with oral clindamycin 450 mg three times daily for seven days. Skin biopsy was done after dermatology consultation. In light of the strong clinical suspicion of STSS, the patient was started on IV immunoglobulin (110 gm). The patient remained on the ventilator on minimal ventilator settings. Skin biopsy was obtained, followed by hematoxylin and eosin stain of the tissue section exhibiting small supra-basilar bulla minimal intraepidermal inflammation and mild perivascular thin-walled dermal inflammation. The studies were sent for dermatopathological evaluation, which resulted in no evidence of an active inflammation dermatosis and negative direct immunofluorescence study. Following consultation with a surgeon, the tissue was extensively debrided due to the formation of necrotic lesions, and additional arrangements for amputating the left foot's dry gangrene were made. Figure 7 and Figure 8 present the biopsy images, showing some surface blood and inflammation, reactive surface squamous epithelium, focus of the dermal perivascular inflammation, and bulla of the bullous impetigo histology.

## Discussion

Despite being two separate illnesses, TSS and bullous impetigo can both be brought on by the same pathogen, most frequently *Staphylococcus aureus* [3]. *S. pyogenes* can occasionally act as a causal agent, as it did in this instance.This example serves as a reminder of the importance of conducting a cultural assessment in each instance of bullous impetigo or cellulitis, especially if diabetes is a concomitant condition. According to the WHO, it causes more than 500,000 fatalities every year and has a yearly incidence of 3.5 cases of invasive GAS per 100,000 people [1,2,3], with a fatality rate of 20-60%. In addition, according to the 2023 Centers for Disease Control and Prevention (CDC) data, there is an increase in the incidence of invasive GAS infections in various US regions. 

The pathogenesis of a GAS infection is complex and multifactorial, involving interactions between the host and bacterial virulence factors, leading to resistance in innate and adaptive immune responses [4,5]. GAS produces various virulence factors, which are involved in both colonization and invasive diseases. The virulence factors are broadly divided into M protein and non-M protein antigens. The virulence of M protein is primarily due to their immunomodulatory effects [6]. An antigen is typically taken in by an antigen-presenting cell, processed, expressed on the cell surface in conjunction with class II MHC, and identified by a T-cell receptor that is specific to that antigen. The result is the activation of polyclonal T cells. Superantigens do not need to be processed by antigen-presenting cells; instead, they interact directly with the invariant region of the class II MHC molecule. In TSS patients, up to 20% of the body's T-cells can be activated at once. This polyclonal T-cell population causes a cytokine storm, which results in the development of a multisystem disease.

In STSS, the skin is the most common portal of entry, with soft tissue infections in at least 80% patients on presentation, as seen in this case. Cutaneous signs may manifest as local erythema, cellulitis, bullous lesions, necrotizing fasciitis, and gangrene. This type of soft tissue involvement is rare in STSS. Rapid progression of local signs and development of systemic manifestations of TSS occur within 48-72 hours and are associated with increased mortality [6,7,8]. The diagnostic criteria for STSS, set forth by the National Notifiable Disease Surveillance System include hypotension with systolic blood pressure (SBP) of less than or equal to 90 mmHg and multiorgan involvement characterized by one or more of the following: renal impairment (Cr > 2mg/dl), coagulopathy (platelets < 100,000, DIC), hepatic involvement as transaminitis, acute respiratory distress syndrome (ARDS), desquamating erythematous macular rash, necrotizing fasciitis, myositis, or gangrene, with laboratory diagnosis confirming isolation of GAS [9].

*S. pyogenes *has more than 200 different serotypes that have been identified based on differences in the M protein-coding regions. By forming complexes between platelets and leukocytes, certain serotypes have been found to have a higher pro-inflammatory capacity [4,9]. Future research on the serotypes of this agent in clinical settings may aid in identifying infections with the highest risk of consequences, enabling tighter monitoring of these individuals.

The treatment for TSS requires immediate medical attention and often necessitates hospitalization typically involving IV antibiotics, immunomodulatory therapies, fluid resuscitation, and supportive care. Although bullous impetigo is not typically associated with the development of STSS, there have been rare instances where individuals with bullous impetigo, particularly when the infection is extensive or severe, may experience symptoms suggestive of STSS. It is important to note that these are general differences, and the presentation of both conditions can vary.

This patient was managed with broad-spectrum antibiotics, de-escalating with sensitivity, and due to the clinical presentation of STSS, the patient was managed with IV immunoglobulin. Due to the extensive skin involvement, the patient was transferred to a burn unit for further management.

It is not entirely known how STSS causes disease. Nevertheless, the mortality of this organism may be influenced by the host's response to streptococcal infection and circulating enterotoxins. A combination of immunoglobulin and hemoperfusion therapy may be beneficial for treating severe instances in patients who have been admitted to the intensive care unit (ICU) and have multiple organ failure. The use of adsorption cartridges in STSS may introduce a new therapeutic approach in severe cases, despite their lack of extensive documentation. Their use in managing proinflammatory mediators, which lead to organ malfunction and cellular damage [8], may be crucial in lowering mortality rates.

## Conclusions

STSS is considered a rare but serious illness with variable mortality, and its exact mechanism remains obscure. In summary, this case report highlights an infrequently reported association between bullous impetigo and STSS. The coexistence of these two distinct entities certainly raises intriguing queries regarding potential triggering factors or shared pathophysiology. Given its life-threatening nature, clinicians should always keep *Streptococcus* species in the differential diagnosis of toxic shock to prevent severe complications, such as death. Further research and studies can reveal underlying mechanisms that should aid in improved diagnosis, outcome, and development of disease-specific treatment guidelines.
